# Dissecting the Superior Drivers for the Simultaneous Improvement of Fiber Quality and Yield Under Drought Stress Via Genome‐Wide Artificial Introgressions of *Gossypium barbadense* into *Gossypium hirsutum*

**DOI:** 10.1002/advs.202400445

**Published:** 2024-07-10

**Authors:** Bei Han, Wenhao Zhang, Fengjiao Wang, Pengkai Yue, Zhilin Liu, Dandan Yue, Bing Zhang, Yizan Ma, Zhongxu Lin, Yu Yu, Yanqin Wang, Xianlong Zhang, Xiyan Yang

**Affiliations:** ^1^ National Key Laboratory of Crop Genetic Improvement Huazhong Agricultural University Wuhan 430070 China; ^2^ Hubei Hongshan Laboratory Wuhan 430070 China; ^3^ Cotton Institute Xinjiang Academy of Agriculture and Reclamation Science Shihezi 832000 China; ^4^ College of Life Sciences Tarim University Alar 843300 China

**Keywords:** cotton, drought stress, DRR1, DRT1, QTL

## Abstract

Global water scarcity and extreme weather intensify drought stress, significantly reducing cotton yield and quality worldwide. Drought treatments are conducted using a population of chromosome segment substitution lines generated from E22 (*G. hirsutum*) and 3–79 (*G. barbadense*) as parental lines either show superior yields or fiber quality under both control and drought conditions. Fourteen datasets, covering 4 yields and 4 quality traits, are compiled and assessed for drought resistance using the drought resistance coefficient (DRC) and membership function value of drought resistance (MFVD). Genome‐wide association studies, linkage analysis, and bulked segregant analysis are combined to analyze the DR‐related QTL. A total of 121 significant QTL are identified by DRC and MFVD of the 8 traits. CRISPR/Cas9 and virus‐induced gene silencing techniques verified *DRR1* and *DRT1* as pivotal genes in regulating drought resistant of cotton, with hap^3‐79^ exhibiting greater drought resistance than hap^E22^ concerning *DRR1* and *DRT1*. Moreover, 14 markers with superior yield and fiber quality are selected for drought treatment. This study offers valuable insights into yield and fiber quality variations between *G. hirsutum* and *G. barbadense* amid drought, providing crucial theoretical and technological backing for developing cotton varieties resilient to drought, with high yield and superior fiber quality.

## Graphical abstract

Here, the study identifies QTL and genes significantly enhancing cotton yield and fiber quality under drought stress through QTL mapping of allotetraploid interspecific chromosomal segment substitution line populations, as well as functional analysis of genes using techniques such as CRISPR/Cas9 and virus‐induced gene silencing techniques. Aggregate analysis is used to explore the relationship between drought resistance, yield, and fiber quality.

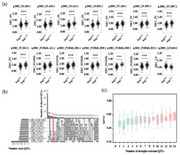

## 1 Introduction

Cotton is a vital economic crop worldwide and is crucial in national economies. The *Gossypium* genus encompasses 52 identified species, with *Gossypium hirsutum* and *Gossypium barbadense* emerging as the two primary tetraploid cultivated cotton species in current production.^[^ ^1^ ^]^ These two plant species collectively contribute ≈90% and 10%, respectively, of the global cotton yield.^[^ ^2^ ^]^ *G. hirsutum*, which is widely cultivated worldwide, is characterized by robust adaptability, high yield, and moderately good fiber quality. Conversely, *G. barbadense*, known for its long‐staple fibers, exhibits exceptional fiber quality but a comparatively lower yield than *G. hirsutum*.^[^ ^3^ ^]^ Unraveling the disparities between *G. hirsutum* and *G. barbadense* and amalgamating their advantages in cultivating high‐yielding varieties with superior fiber quality are significant challenges. Constructing artificial introgression populations, such as chromosomal segment substitution lines (CSSLs), to introduce favorable allelic variations into *G. hirsutum*, is an ideal solution.^[^ ^4^, ^5^, ^6^ ^]^ This approach has also been widely employed in addressing other species, such as maize,^[^ ^7^ ^]^ rice,^[^ ^8^, ^9^ ^]^ wheat.^[^ ^10^ ^]^

Although cotton plants are widely grown in different types of soils and climates worldwide, they have not been considered as drought‐resistant plants.^[^ ^11^ ^]^ Drought strongly hinders cotton plants from absorbing moisture from the soil, causing an increase in osmotic potential; abscission of flower buds; reduction in fiber elongation; change in fiber wall thickness, cotton boll size, and fiber quality; and a decrease in total cotton yield.^[^ ^12^, ^13^, ^14^ ^]^ Xinjiang has severe drought problems because it is in semiarid and arid regions with little annual precipitation and extensive evaporation.^[^ ^15^, ^16^ ^]^ Moreover, Xinjiang is the largest cotton‐producing region in China and has a production base of long‐staple uplands and high‐quality commercial cotton.^[^ ^17^, ^18^ ^]^ Therefore, studying the drought resistance of cotton germplasm resources and identifying alleles conducive to drought resistance are highly practical.

Drought resistance is a quantitative genetic trait dominated by microeffect polygenes.^[^ ^19^, ^20^, ^21^ ^]^ At present, many countries have carried out substantive research on plant resistance physiology and biotechnology under drought stress. As early as 1989, Savage et al.^[^ ^22^ ^]^ demonstrated that improving the ability of roots to absorb water can alleviate drought stress. With the emergence of new sequencing technologies and the improvement of crop reference genomes, the mining of drought resistance quantitative trait loci (QTL) has gradually developed from linkage analysis using genetic maps in the early stage to genome‐wide association studies (GWAS) using population resequencing. Most drought‐related studies primarily utilize constructed F_2_ or natural populations to explore drought‐tolerant loci within *G. hirsutum*, leading to limited utilization of the advantages offered by different cotton species.^[^ ^13^, ^23^, ^24^, ^25^ ^]^ Recent studies have indicated that the identification and utilization of inter‐specific introgression segments, particularly from *G. barbadense*, through the use of inter‐specific segregating populations constructed by crossing *G. hirsutum* × *G. barbadense*, can effectively improve cotton yield, quality, and related resistance traits.^[^ ^26^, ^27^, ^28^, ^29^, ^30^, ^31^, ^32^ ^]^ However, Studies focusing on the loci or introgression segments that can concurrently improve both fiber yield and quality under drought stress conditions are rarely reported.

In this study, we employed E22 (*G. hirsutum*) and 3–79 (*G. barbadense*) to construct a population of CSSLs comprising 319 accessions. Phenotypic data for 14 traits were collected over multiple years of field‐based drought stress treatment conducted during the flowering and boll stages in Xinjiang. The QTL and candidate genes related to cotton drought resistance were identified via GWAS, linkage analysis, and bulked segregant analysis (BSA). Furthermore, the biological functions of the candidate genes *DRR1* (encoding a heat shock protein 20) and *DRT1* (encoding a PLETHORA1‐like transcription factor) were validated using the clustered regularly interspaced short palindromic repeats (CRISPR)‐CRISPR‐associated protein 9 (Cas9) system and virus‐induced gene silencing (VIGS) techniques, highlighting that the hap^3‐79^ haplotype exhibits stronger drought resistance than hap^E22^ in terms of *DRR1* and *DRT1*. Additionally, through multisite aggregation analysis and analysis of the membership function value of drought resistance (MFVD), the relationship between yield and fiber quality under drought treatment were explored. The use of *G. hirsutum* and *G. barbadense* interspecific CSSLs effectively disrupted the linkage burden, synergistically enhancing cotton yield and fiber quality under drought conditions. This study offers novel insights for improving cotton drought resistance and optimizing quality.

## 2 Results

### 2.1 *G. hirsutum* E22 and *G. barbadense* 3–79 Maintain Superior Yield or Fiber Quality Characteristics Under Different Water Conditions

**Figure 1:**
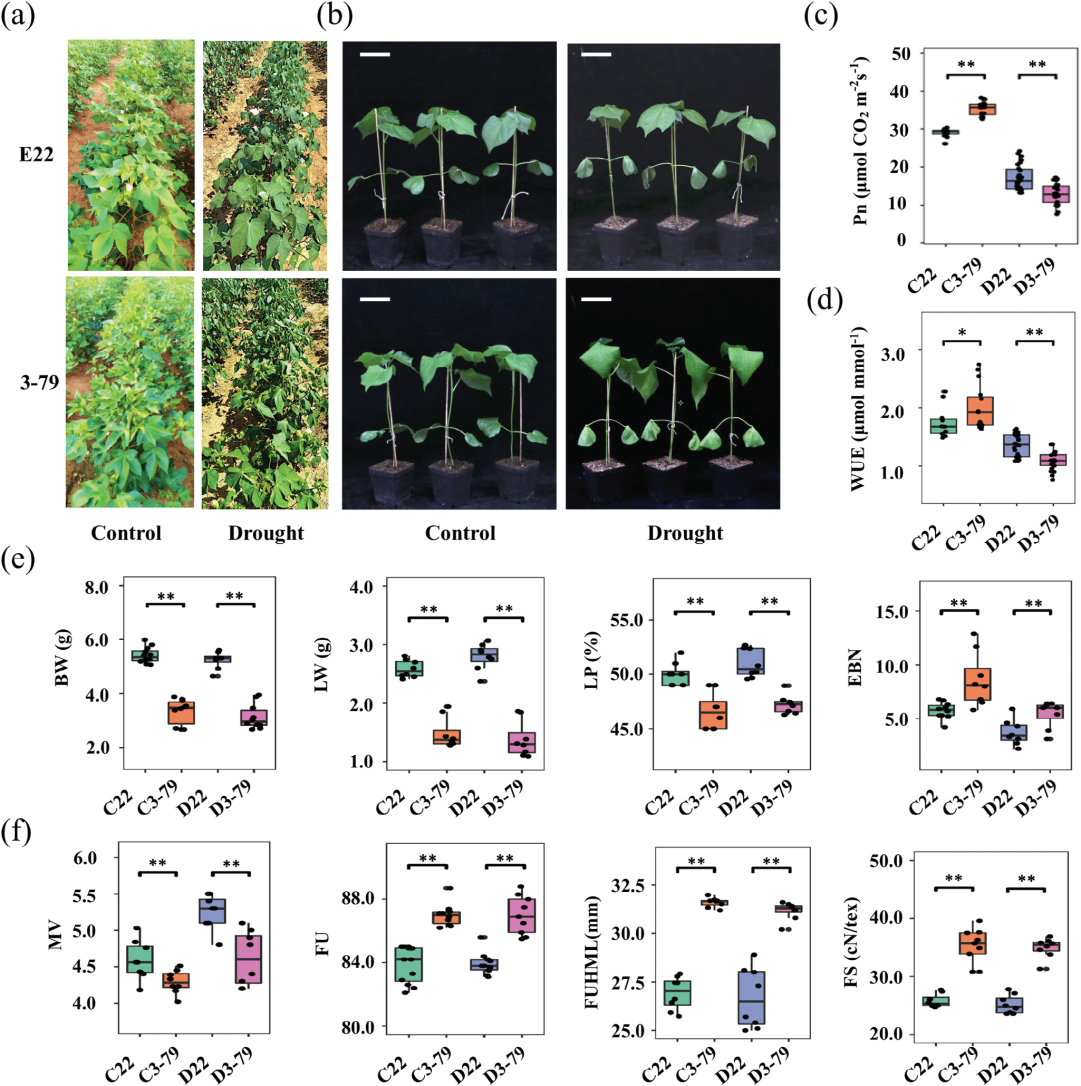
E22 and 3–79 maintain their superior yield or fiber quality characteristics under different water conditions. a,b) Phenotypes of E22 and 3–79 plants planted in the field and greenhouse. c,d) Field photosynthesis‐related indices, including the Pn and WUE, of the functional leaves from E22 and 3–79 were measured in the field. e) Compared with 3–79, E22 exhibited greater phenotypic values, such as BW, LW, and LP, under control and drought conditions. f) Compared with E22, 3–79 exhibited better phenotypes for MV, FU, FUHML, and FS under control and drought conditions. C22 and D22 represent E22 under normal irrigation conditions and drought conditions, and C3‐79 and D3‐79 represent 3–79 under normal conditions and drought conditions, respectively. At least eight biological replicates from each group were analyzed. Asterisks (* and **) indicate significant differences at a *p*‐value < 0.05 and *p*‐value < 0.01, respectively, as determined by one‐way ANOVA followed by a *t*‐test.

Emian 22 (E22, recurrent parent) is a *G. hirsutum* cultivar bred in Hubei Province, China, with high lint yield and moderate fiber quality, while 3–79 (nonrecurrent parent) is the standard line of *G. barbadense* for genetic research and exhibits excellent fiber quality. The two varieties were grown in the field and greenhouse under different water conditions. 3–79 exhibited stronger growth than E22 under normal watering conditions. However, the leaves of 3–79 plants wilted, while those of E22 did not wilt under drought stress in the field or greenhouse (**Figure** **1**a,b). Photosynthesis‐related indicators (photosynthesis rate(Pn); and water use efficiency(WUE)) were determined during the flowering and boll stages in the field. These indicators were adversely affected in both E22 and 3–79 under drought conditions. However, these parameters were greater in 3–79 than in E22 under normal irrigation conditions but lower in 3–79 than in E22 under drought stress (Figure 1c,d), indicating that E22 maintained photosynthesis to a certain extent under drought stress. The yield and fiber quality of the plants were determined over the years. As expected, compared with 3–79, E22 exhibited superior yield traits in terms of boll weight (BW), lint weight (LW), lint percentage (LP) (Figure 1e). Conversely, 3–79 outperformed E22 in terms of fiber uniformity (FU), fiber upper half mean length (FUHML), fiber strength (FS), and micronaire value (MV) under normal conditions (Figure 1f). Moreover, these two varieties retained their superior characteristics even under drought conditions, although 3–79 showed wilting during the different stages (Figure 1a,b). Overall, E22 exhibited remarkably high yield potential, whereas 3–79 exhibited excellent fiber quality under different water conditions. The complementary strengths of these two parents render them suitable for the simultaneous improvement of fiber quality and yield in challenging environments.

### 2.2 Analysis for Phenotypic Variation

**Figure 2:**
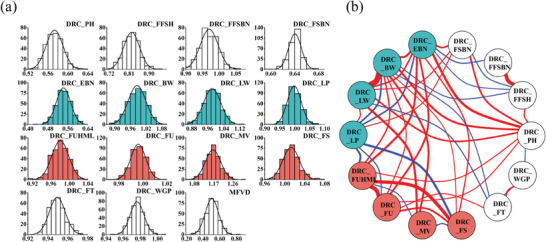
The frequency distribution and correlation network of 14 DRC traits and MFVD in CSSLs. a) The frequency distributions of 14 DRC traits and MFVD in CSSLs. b) Correlation network between traits. The red line indicates a positive correlation (*p*‐value < 0.05), the blue line indicates a negative correlation (*p*‐value < 0.05), and the thickness of the line indicates the strength of the correlation.

Using *G. hirsutum* E22 as the recurrent parent and the *G. barbadense* genetic standard line 3–79 as the nonrecurrent parent, an interspecific CSSL population spanning the entire cotton genome was meticulously developed through extensive hybridization and backcrossing over several years.^[^ ^33^ ^]^ Field trials in which drought stress was imposed were subsequently carried out in Xinjiang to assess the drought resistance of this population. Fourteen traits, including plant height (PH), flowering time (FT), whole growth period (WGP), first fruit spur height (FFSH), first fruit spur branch number (FFSBN), fruit spur branch number (FSBN), EBN, BW, LW, LP, FUHML, FU, FS and MV traits, of the CSSLs were evaluated through two years of experiments involving two treatments (normal watering and drought treatment conditions) in Xinjiang. The DRC is an important indicator used to reflect the degree of drought stress. The histogram of the DRC frequency distribution for each trait showed a normal distribution (**Figure** **2**a). The 14 phenotypes of the 319 accessions under different treatment conditions were estimated separately with the best linear unbiased predictions (BLUPs) based on two years of experimental data. Table S1 (Supporting Information) shows the mean (mean), maximum (max), minimum (min), standard deviation (SD), and coefficient of variation (CV) of the 14 traits under the two conditions. The statistical analysis of normal watering and drought conditions data showed that all the agronomic traits were strongly affected by drought treatment. For example, the mean WGP varied from 127.870 to 137.103 days, with an average value of 131.665 days under normal watering conditions, while under drought treatment, it varied from 125.427 to 133.695 days, with an average value of 128.522 days under water stress. The PH was 61.723–70.581 cm (average 65.507 cm) under normal circumstances and 33.615–41.464 cm (average 37.541 cm) under drought treatment. FSBN ranged from 7.685 to 7.991 (average 7.804) under normal watering conditions, and FSBN ranged from 4.766 to 5.285 (average 4.999) under drought treatment. The average WGP, PH, and FSBN decreased by 2.387%, 42.691%, and 35.951%, respectively, under drought treatment. In addition, the yield traits EBN, BW, and LW were reduced by 46% (from 5.539 to 2.997), 1.47% (from 5.293 g to 5.215 g), and 1.85% (from 2.420 g to 2.375 g), respectively, but LP was slightly greater.

Traits related to fiber quality were also adversely affected by the water deficit. The FUHML decreased from 26.973 to 26.427 mm, and the FU decreased from 83.829% to 83.507%. In addition, the MV decreased from B to C (4.633 to 5.405) due to drought stress, but the FS increased from 25.985 to 26.301 cN tex^−1^. To determine whether drought stress affects phenotyping, we performed ANOVA and found that all 14 phenotypes reached significant levels.

To assess the comprehensive drought resistance and drought tolerance levels of cotton plants, we combined the complementary advantages of parental lines in terms of drought resistance, yield, and fiber quality traits, as well as the high heritability of yield and fiber quality traits. A previously reported method was used to analyze the drought resistance coefficients of eight crucial traits via the MFVD (Table S2, Supporting Information). The evaluation of comprehensive drought resistance was based on the magnitude of the MFVD values, where larger MFVD values indicated stronger drought resistance. A frequency histogram illustrated a normal distribution of MFVD values (Figure 2a). The drought tolerance of the population materials was categorized into five levels based on the MFVD values (Table S2, Supporting Information). Level I indicated a high degree of drought sensitive (9 lines), Level II represented moderate drought sensitive (68 lines), Level III indicated non‐drought tolerance (150 lines), Level IV represented moderate drought tolerance (83 lines), and Level V represented a high degree of drought tolerance (9 lines).

The correlation between the DRC of each trait reflects the interrelationships between traits and might be regulated by similar genetic factors. Pearson correlation analysis between 14 traits of the DRC showed that many traits were correlated (Table S3, Supporting Information). The correlation analysis of 14 traits revealed 91 pairs with correlation coefficients ranging from −0.312 to 0.912; 42 pairs had significant correlations (*p*‐value < 0.05, *R* ^2^ > 0.1 or *R* ^2^ < −0.1) (Figure 2b), including the trait pairs DRC_BW and DRC_LW and the trait pairs DRC_LW and DRC_LP, each of which had a significant positive correlation. The four fiber quality traits (DRC_FU, DRC_FUHML, DRC_FS, and DRC_MV) exhibited strong positive phenotypic and genotypic correlations with each other; in addition to DRC_MV, which was negatively correlated with the others, all the other traits were significantly positively correlated.

### 2.3 GWAS, Linkage Analysis and BSA‐seq

To fully exploit the complementary advantages of the two parents and analyze the genetic mechanism underlying the difference in drought tolerance between the CSSL population, the reported resequencing data of 321 accessions (319 CSSL+2 parents) were integrated and analyzed. A total of 22103084 single nucleotide polymorphisms (SNPs) were obtained after mapping to the reference genome TM‐1 using Sentieon software. A total of 1309847 high‐quality SNPs were obtained for subsequent GWAS by filtering out scaffolds, those with an MAF≤0.05, and those with missing values ≥0.5.

**Figure 3:**
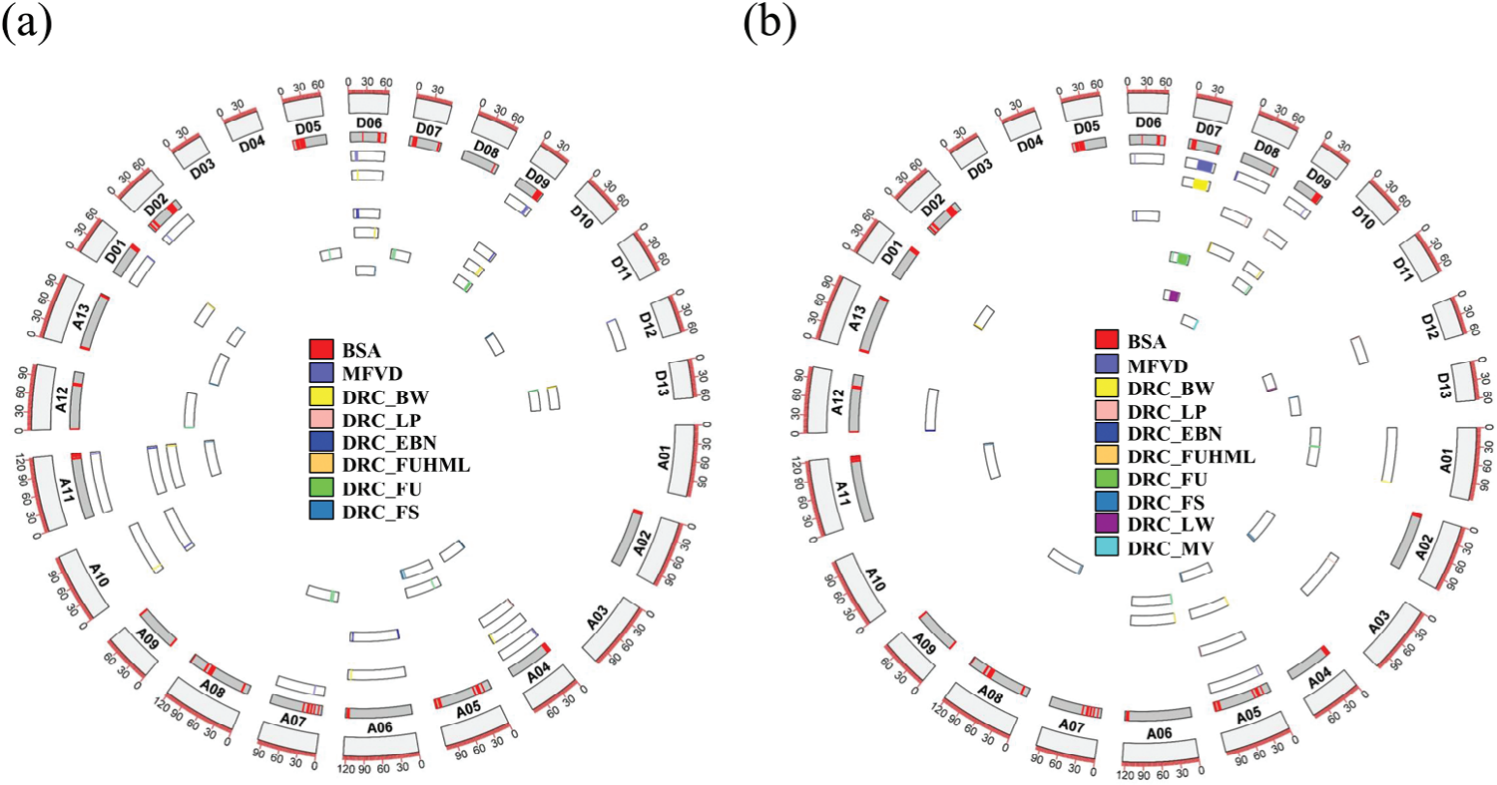
Summary of QTL identified via GWAS, linkage analysis, and BSA. a) Identification of QTL based on GWAS and BSA. b) Identification of QTL based on linkage analysis and BSA.

The obtained SNPs were subjected to GWAS on the basis of the DRC of 8 traits (DRC_BW, DRC_LW, DRC_LP, DRC_EBN, DRC_FUHML, DRC_FU, DRC_FS, DRC_MV) and MFVD via a mixed linear model using FastLMM software. In total, 52 QTL (12 for MFVD) were identified among the 9 traits on 21 chromosomes under the suggested association threshold (*p*‐value = 7.63487E^−07^), including 40 QTL mapped from 8 DRC traits (**Figures** **3**a, S1, Table S4, Supporting Information). We also found that multiple traits were associated with the same QTL. The results showed that DRC_EBN and DRC_BW were in the same QTL interval on chromosomes A06, A10, and D06; DRC_FU, DRC_FS, and DRC_FUHML were identified in the same QTL on chromosomes A11, D09 and D13; and DRC_FS and DRC_LP were colocalized in the A04 interval. This result was strongly correlated with the Pearson correlation coefficient of the trait.

To assess the importance of valuable genetic loci for drought resistance in interspecific hybrid populations of cotton breeding, a genotype map was reconstructed using introgressed chromosome recombination fragments for linkage analysis. Nine traits were analyzed using the RSTREP‐LRT‐ADD mapping method in QTL IciMapping software, and a total of 34 QTL were identified on 26 chromosomes (Table S4, Supporting Information), of which 26 were identified by DRC of 8 major yield and quality traits (DRC_BW, DRC_LW, DRC_LP, DRC_EBN, DRC_FUHML, DRC_FU, DRC_FS, and DRC_MV) (Figure 3b). Most remarkably, DRC_EBN, DRC_FUHML, and MFVD were found to be associated with the same QTL on chromosome D06 (D06: 8907423–8980455); DRC_BW, DRC_LW, and DRC_FU were found to be colocalized within the 28.9 Mb interval of chromosome D07 (D07: 24636085–53588497); and DRC_FUHML and MFVD were found to be associated with the same QTL on chromosome D09 (D09: 40466793–40830748). The overall QTL distribution of the 34 QTL was not significantly different between the two subgenomes (A: 15 QTL, D: 19 QTL).

To accurately determine the position of the QTL from multiple perspectives, we combined the characteristics of the CSSL populations and used the MFVD method to screen 30 drought‐resistant lines and 30 drought‐sensitive lines, dividing them into two pools (Table S2, Supporting Information). The QTL interval was determined by calculating the ΔSNP‐index between the two pools. We selected 2 different molecular markers from the two pools, totaling 11004912 markers. Using the R package QTLseqr, we calculated the SNP‐index and ΔSNP‐index of the offspring pools separately using a sliding window of 1.5 Mb. By setting the threshold line at the 99% confidence level to determine significant association intervals, sixty‐three QTL were identified with a range of 20 kb‐13 Mb (Figure S2, Table S4, Supporting Information). Among them, 36 QTL were co‐located according to GWAS, and 7 intervals were co‐located according to linkage analysis.

A total of 121 QTL were identified using three different methods, and 39 QTL were co‐located by at least two methods, of which 6 QTL were identified using all three methods (Table S4, Supporting Information).

### 2.4 DRR1 Positively Regulates Drought Tolerance in Cotton

The response of plants to drought stress is regulated by many genes; therefore, obtaining expression profile data related to cotton drought stress can greatly improve the screening efficiency of candidate genes. To identify drought resistance genes more accurately, we combined the LD attenuation distance and selected intervals of 300 kb upstream or downstream of the highlighted SNPs as the screening range for the candidate genes. We screened a total of 93 differentially expressed candidate genes associated with drought stress within nine colocalization candidate QTL intervals (Table S5, Supporting Information) by analyzing the expression profile data of all the genes in the QTL candidate interval in the drought‐sensitive line (M048) and drought‐resistant line (M307) and annotating homologous *Arabidopsis* genes.

**Figure 4:**
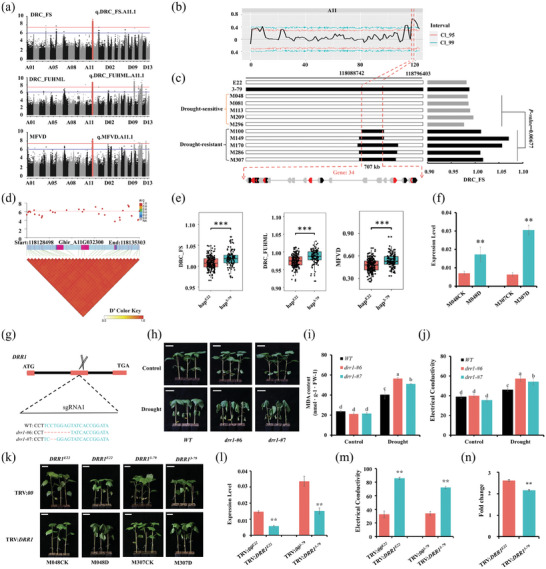
Mapping and biological function of the candidate gene *DRR1*. a) GWAS results for DRC_FS, DRC_FUHML, and MFVD; red dots indicate SNPs in the A11 candidate interval. b) BSA in A11 chromosomal localization. c) Linkage analysis results for DRC_FS. d) Association analysis of the genetic variation in *DRR1* with DRC_FS and the pattern of pairwise LD of the associated SNPs in *DRR1*. e) Plants with the hap^3‐79^ haplotype presented significantly greater levels of DRC_FS, DRC_FUHML, and MFVD than those with the hap^E22^ haplotype in the CSSLs. f) Relative expression levels of *DRR1* in M048 (hap^E22^) and M307 (hap^3‐79^). g) Schematic diagram of the structure of the *DRR1* mutant plant; the black‒red box indicates exons, and the black lines indicate introns. The scissors represent the location of the designed sgRNA. h) Both types of *DRR1* exhibited drought stress sensitivity compared to that of the WT. There was no significant difference under normal conditions. i,j) MDA content and relative electrical conductivity of WT and *DRR1* plants under control and drought stress conditions. Error bars are calculated as standard deviations of at least 3 biological replicates, and different lowercase letters above the columns indicate significant differences between columns (Duncan's multiple comparisons, *p*‐value < 0.05). k) TRV:*DRR1* exhibited varying levels of sensitivity to drought stress after its expression was reduced in M048 (hap^E22^) and M307 (hap^3‐79^). l) RT‒qPCR analysis of the relative expression levels of *DRR1* to determine the silencing effect on M048 (hap^E22^) and M307 (hap^3‐79^). m) Relative electrical conductivity of separated leaves of M048 (hap^E22^) and M307 (hap^3‐79^). n) The fold change in the relative electrical conductivity of leaves before and after silencing the candidate gene *DRR1* in M048 (hap^E22^) and M307 (hap^3‐79^). The values are presented as the means ± SD from at least three independent replicates, and asterisks (** and ***) indicate significant differences at *p*‐value < 0.01 and *p*‐value < 0.001, respectively, based on the *t*‐test.

We found a hot spot on chromosome A11. The QTL q.DRC_FUHML.A11.1, q.DRC_FS.A11.1 and q.MFVD.A11.1 were colocalized in this hotspot with three traits (DRC_FUHML, DRC_FS, and MFVD) according to the GWAS results (**Figure** **4**a). The highlighted SNPs in this region were A11‐118429839, A11‐118276349, and A11‐118420181. Combining the LD block results, this interval was determined to be within A11: 118034834–118797814. Moreover, a QTL that significantly exceeded the threshold in A11 was identified in two pools through MFVD screening. The identified QTL was located within a 5 Mb interval (A11:117108146‐122110218) (Figure 4b, Table S4, Supporting Information). Moreover, the QTL was also identified in the linkage analysis of DRC_FS and MFVD, and the localization interval was 707 kb (A11:118088742‐118796403) (Table S4, Supporting Information). Further analysis indicated that the drought‐resistant group (e.g., M100, M49, M170, M286, and M307) within this interval contained introgressed segments from 3–79, while the drought‐sensitive group (e.g., M048, M081, M113, M209, and M296) did not contain introgressed segments (Figure 4c). In summary, we localized this hotspot in the A11 chromosome 707 kb interval (A11: 118088742–118796403).

Thirty‐four genes were detected within the 707 kb interval, of which 6 differentially expressed candidate genes associated with drought stress were identified (Figure 4c, Table S5, Supporting Information). Among them, a gene *DRR1* (drought resistance regulator 1, Ghir_A11G032300, chromosomal interval: A11:118130498‐118133303) which encoded a heat shock protein, exhibited 14 variation loci on its promoter and CDS (promoters: G/C (−131), C/A (−142), C/T (−280), G/A (−512), C/T (−546), T/C (−549), G/A (−678), A/G (−686), A/T (−970), A/G (984) and C/T (−1039); CDS: A/G (+89), G/A (+1516), G/A (+1584)). Haplotype analysis of *DRR1* revealed that the SNPs in the gene were tightly linked (R^2^>0.8) and contained only two major haplotypes (Figure 4d). We performed haplotype analysis on five lines in the drought‐resistant and drought‐sensitive pools and found that the haplotype of the drought‐resistant lines was hap^3‐79^ and that the haplotype of the drought‐sensitive lines was hap^E22^. Analysis of the two haplotypes using phenotypic values showed that hap^3‐79^ had significantly greater DRC_FS, DRC_FUHML, and MFVD than hap^E22^ (Figure 4e). Moreover, using real‐time quantitative PCR (RT–qPCR), we determined that the relative expression of *DRR1* significantly differed in the CSSL lines with different haplotypes, M048 (hap^E22^) and M307 (hap^3‐79^) before and after drought treatment, and the line M307 (hap^3‐79^) exhibited a greater expression level than the line M048 (hap^E22^) following drought conditions (Figure 4f).

The CRISPR/Cas9 gene editing technique was used to create *drr1* mutant plants using a single sgRNA (Figure 4g). The cotyledons and leaves of plants from two types of *drr1* mutants (*drr1‐#6* and *drr1‐#7*) exhibited greater wilting and withering than those of the wild type (WT) under drought stress, while no significant differences were observed under normal conditions (Figure 4h). The levels of malondialdehyde (MDA) and relative electrical conductivity, two crucial physiological indicators of cell damage, were significantly greater in the *drr1* plants than in the wild type after drought, indicating a greater extent of stress injury (Figure 4i,j).

To further investigate the functional mechanisms of the candidate gene *DRR1* in response to drought stress in different haplotypes (hap^E22^ and hap^3‐79^), we utilized VIGS technology to knock down the candidate gene *DRR1* in the CSSL lines with different haplotypes, M048 (hap^E22^) and M307 (hap^3‐79^). The recombinant pTRV2 vector was constructed to include the candidate gene TRV:*DRR1*. The pTRV2 vector (TRV:*00*) or *GhCLA* (TRV:*CLA*) fragment without DNA insertion was used as a control or VIGS efficiency indicator. The silencing effect was detected after the expected complete photobleaching phenotype appeared on true leaves of the TRV:*CLA* plants (Figure S3a,b, Supporting Information). The RT‒qPCR results showed that the expression levels of the corresponding gene *DRR1* in the TRV:*DRR1*‐silenced lines were significantly lower than those in the TRV:*00* plants (Figure 4l). After drought treatment, both the M048 (hap^E22^) and M307 (hap^3‐79^) lines exhibited more severe wilting in TRV:*DRR1* plants than in TRV:*00* leaves, indicating that silencing *DRR1* increased the sensitivity of cotton plants to drought stress (Figure 4k). The results of the relative electrical conductivity analysis of the plant leaves indicated more severe damage to TRV:*DRR1* in both M048 (hap^E22^) and M307 (hap^3‐79^) plants (Figure 4m). Taken together, these findings indicated that *DRR1* is vital in enhancing drought resistance in cotton plants across different genotypes. Furthermore, after the candidate gene *DRR1* was silenced, haplotype M048 (hap^E22^) exhibited more severe leaf wilting than haplotype M307 (hap^3‐79^). To illustrate this difference in drought resistance, we calculated the fold change in leaf relative electrical conductivity between M048 (hap^E22^) and M307 (hap^3‐79^) before and after silencing the candidate gene *DRR1* (Figure 4n). The results showed that after silencing the candidate gene *DRR1*, the fold change in M048 (hap^E22^) was significantly greater than that in M307 (hap^3‐79^), indicating that, compared with hap^E22^, *DRR1* is prominent in regulating drought stress in hap.^3‐79^

### 2.5 DRT1 is a Positive Regulator of Cotton Drought Tolerance

**Figure 5:**
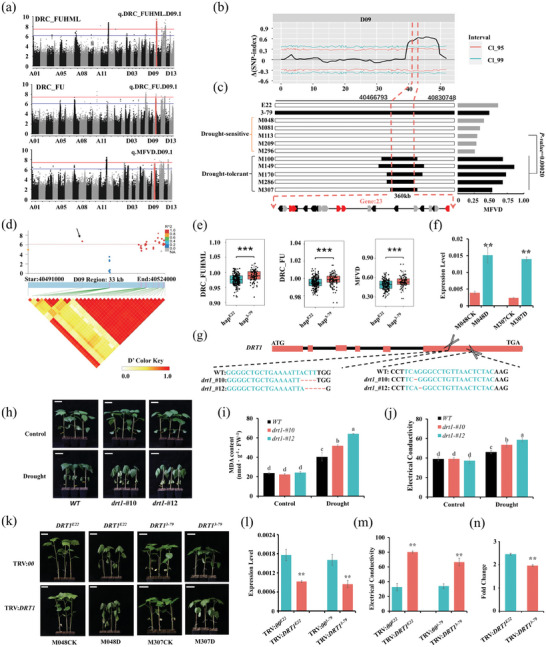
Mapping and biological function of the candidate gene *DRT1*. a) GWAS results for DRC_FUHML, DRC_FU, and MFVD; red dots indicate SNPs in the D09 candidate interval. b) BSA and c) linkage analysis results for D09 chromosomal localization. d) Association analysis of the genetic variation in *DRT1* with DRC_FUHML and the pattern of pairwise LD of the associated SNPs in the candidate interval. The black arrows indicate nonsynonymous mutations located in the *DRT1* exon. e) Plants with the hap^3‐79^ haplotype presented significantly greater levels of DRC_FUHML, DRC_FU, and MFVD than those with the hap^E22^ haplotype in the CSSLs. f) Relative expression levels of *DRT1* in M048 (hap^E22^) and M307 (hap^3‐79^). g) Schematic diagram of the structure of the *DRT1* mutant plant; the red box represents exons, and the black lines represent introns. Two scissors represent the location of the designed sgRNA. h) Both *DRT1* mutants exhibited drought stress sensitivity compared to that of the WT. No significant difference was observed under normal conditions. i) MDA and j) relative electrical conductivity contents of WT and *DRT1* plants under control and drought stress conditions. Error bars are calculated as standard deviations of at least 3 biological replicates, and different lowercase letters above the columns indicate significant differences between columns (Duncan's multiple comparisons, *p*‐value < 0.05). (k) TRV:*DRT1* exhibited varying levels of sensitivity to drought stress after its expression was reduced in M048 (hap^E22^) and M307 (hap^3‐79^). l) RT‒qPCR analysis of the relative expression levels of *DRT1* to determine the silence effect on M048 (hap^E22^) and M307 (hap^3‐79^). m) Relative electrical conductivity of separated leaves of M048 (hap^E22^) and M307 (hap^3‐79^). n) The fold change in the relative electrical conductivity of leaves before and after silencing the candidate gene *DRT1* in M048 (hap^E22^) and M307 (hap^3‐79^). The values are presented as the means ± SD from at least three independent replicates, and asterisks (** and ***) indicate significant differences at *p*‐value < 0.01 and *p*‐value < 0.001, respectively, based on the *t*‐test.

In addition, another noteworthy peak was detected on chromosome D09 in the GWAS results. We found that q.DRC_FUHML.D09.1 (‐log_10_(*p*‐value) = 9.42), q.DRC_FU.D09.1 (‐log_10_(*p*‐value) = 7.19) and q.MFVD.D09.1 (‐log_10_(*p*‐value) = 7.28) were colocalized within the same QTL interval according to the results of the DRC_FUHML, DRC_FU and MFVD association analyses (**Figure** **5**a). The highlighted SNPs were D09_40 715 689, D09_40715689, and D09_40996766, and the physical distance between them was <300 kb in the reference genome (Table S4, Supporting Information). This QTL interval was ≈600 kb (D09:40415689‐D09:41015689). Moreover, a significant QTL was detected on chromosome D09 within the interval D09:39607151‐49431632 through the computation of the ΔSNP‐index using QTLseqr software (Figure 5b, Table S4, Supporting Information). Moreover, a significant QTL containing 23 genes was colocalized within an interval of ≈360 kb involute fragments (D09:40466793‐D09‐40830748) when performing linkage mapping of DRC_FUHML and MFVD (Figure 5c, Table S4, Supporting Information). The LODs of these QTL were 5.44 and 2.99. In summary, we located the hotspot in the D09:40466793‐40830748 interval, which contained a total of 23 genes.

Combined with the transcriptome and literature analysis results, 5 differentially expressed candidate genes affected by drought stress were selected within this candidate interval (Table S5, Supporting Information). *DRT1* (drought resistance transcription factor 1, Ghir_D09G012870), a homologous gene with the AT3G20840 in *Arabidopsis*, encoded a PLETHORA‐like transcription factor. Sequence alignment analysis revealed that the *DRT1* gene (D09:40501952‐40504889) contained a significant exon variation (DRC_FUHML: ‐log10 (*p*‐value) = 6.73)) that was a nonsynonymous mutation (G/T) that caused amino acid no.384 to change from the polar amino acid serine (Ser) to the nonpolar amino acid isoleucine (Ile); this SNP could be a potential causal variant that regulates drought tolerance (Figure 5d). The haplotype analysis revealed two major haplotypes, namely hap^E22^ (amino acid: Ile), which was consistent with the female parent (E22), and hap^3‐79^ (amino acid: Ser), which was associated with the male parent (3‐79). Among them, drought‐resistant lines (e.g., M100, M49, M170, M286, and M307) belonged to the hap^3‐79^ haplotype, while drought‐sensitive lines (e.g., M048, M081, M113, M209, and M296) belonged to the hap^E22^ haplotype. Moreover, for DRC_FUHML, DRC_FU, and MFVD in the CSSLs, the abundance of the haplotype hap^3‐79^ was significantly greater than that of hap^E22^ (Figure 5e). Moreover, the relative expression levels of this gene exhibited significant differences in different haplotype lines (M048^E22^ and M307^3‐79^) after drought treatment compared with those in the control treatment (Figure 5f). However, no significant difference was observed between M048 (hap^E22^) and M307 (hap^3‐79^). Therefore, we speculate that this gene may positively regulate drought stress tolerance in cotton.

To investigate the role of *DRT1* in cotton plants under drought conditions, we employed CRISPR/Cas9 technology to knock out the *DRT1* gene. We designed 2 sgRNAs targeting the *DRT1* gene, generating multiple putative transgenic lines in the Jin668 background. Mutation types were identified using Hi‐TOM. The analysis revealed two different base deletions in the *drt1‐#10* and *drt1‐#12* lines, which were further confirmed by Sanger sequencing (Figure 5g). Following drought treatment, both *DRT1* knockout lines exhibited an extremely severe drought stress phenotype characterized by drooping and wilting leaves, whereas the wild‐type plants exhibited greater drought tolerance and less wilting (Figure 5h). Moreover, the knockout lines exhibited significantly greater levels of MDA and relative electrical conductivity than the wild type under drought stress (Figure 5i,j). These findings indicated that the knockout of *DRT1* renders cotton more susceptible to drought stress.

To investigate the function of the candidate gene *DRT1* in different haplotypes (hap^E22^ and hap^3‐79^), the VIGS technique was used to knock down the expression of *DRT1* in the CSSL lines with different haplotypes, M048 (hap^E22^) and M307 (hap^3‐79^). The recombinant pTRV2 vector candidate gene TRV:*DRT1* was constructed. TRV:*00* and TRV:*CLA* were used as controls as previously described. The optimal gene silencing effect was achieved when the true leaves of TRV:*CLA* plants exhibited an obvious photobleaching phenotype (Figure S3a,b, Supporting Information). RT‒qPCR revealed that the expression of TRV:*DRT1* silencing lines was significantly inhibited in both M048 (hap^E22^) and M307 (hap^3‐79^) (Figure 5l). After drought treatment, plants with knocked‐down *DRT1* expression in both M048^E22^ and M307^3‐79^ exhibited more severe wilting than those in TRV:*00* plants, indicating that knocking down *DRT1* expression increased the sensitivity of cotton plants to drought stress (Figure 5k). The relative electrical conductivity in the plant leaves also reflected this result (Figure 5m), further suggesting that *DRT1* is a key gene involved in regulating the cotton response to drought stress. Additionally, compared to the haplotype M307 (hap^3‐79^), the haplotype M048 (hap^E22^) exhibited more severe leaf wilting after knocking down *DRT1* expression. The fold change in relative electrical conductivity in leaves before and after silencing the candidate gene *DRT1* was further calculated for M048 (hap^E22^) and M307 (hap^3‐79^) (Figure 5n). The results indicated that after silencing the candidate gene *DRT1*, the fold change in M048 (hap^E22^) was significantly greater than that in M307 (hap^3‐79^), suggesting that the regulatory effect of hap^3‐79^ on drought stress is greater than that of hap^E22^.

### 2.6 Introgressed Segments of 3–79 to E22 Coordinate Yield and Fiber Quality Under Drought Stress in Cotton

**Figure 6:**
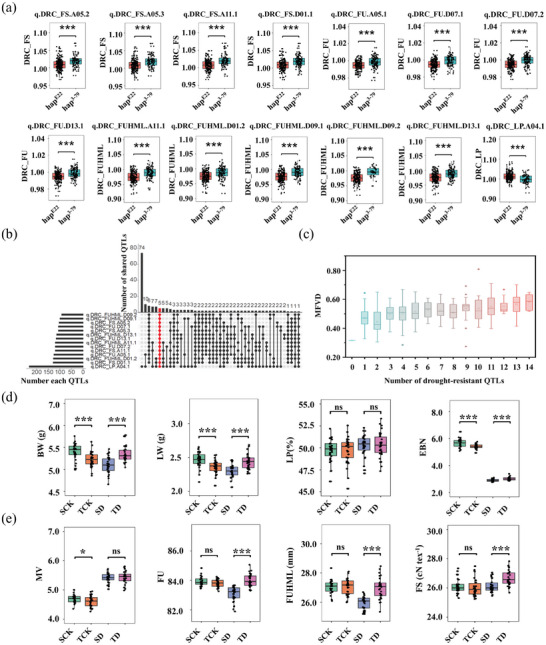
Exploitation of introgressed segments_based SNP markers for evaluating the drought‐resistant CSSLs. a) Box plots for phenotypic values of the haplotypes for highlighted SNPs with 14 colocalized intervals. (***) indicates a significant difference at a *p‐*value < 0.001 according to one‐way ANOVA followed by a *t*‐test. b) Aggregate analysis of 14 drought‐resistant QTL in CSSL populations; red indicates the accessions in which all 14 drought‐resistant QTL were aggregated. c) The number of drought‐resistant sites was positively correlated with the MFVD. d) The drought‐resistant lines exhibited greater yields (LW, BW, and EBN), and e) improved fiber quality (FUHML, FS, and FU) following drought treatment. SCK represents drought‐sensitive ponds under normal conditions, SD represents drought‐sensitive ponds under drought conditions, TCK represents drought‐tolerant ponds under drought conditions, and TD represents drought‐tolerant ponds under drought conditions. Thirty biological replicates from each group were analyzed. (***) indicates a significant difference at a *p*‐value < 0.001 according to one‐way ANOVA followed by a *t*‐test. ns indicates no significant difference.

The E22 genotype exhibited high yield potential under both normal water and drought stress conditions, while the 3–79 genotype exhibited better fiber quality. Conventional breeding through interspecific hybridization has proven challenging because of the difficulty in stably inheriting superior fiber quality traits. Additionally, due to the negative effects of linkage drag on *G. hirsutum* yield, achieving coordinated improvements in both fiber quality and yield between *G. hirsutum* and *G. barbadense* varieties has been difficult. CSSLs offer the greatest potential for breaking down linkage drag and achieving improvements in resistance, yield, and quality. By integrating the characteristics of the parental genotypes, we analyzed and located the results for traits related to yield and fiber quality correlation under drought conditions. The DRCs for both yield and fiber quality traits were mapped for 66 QTL. Twenty‐nine QTL were identified by at least two methods that showed associations with DRC_yield traits and DRC_quality traits. Due to the wide interval between linkage analysis and BSA, subsequent analyses were conducted using the GWAS results, which facilitated the identification of correlations between candidate genes. The haplotypes of 29 QTL were categorized based on the highlighted SNPs (the most significant SNPs) identified by GWAS. A genotype consistent with 3–79 was denoted as hap,^3‐79,^ and a genotype consistent with E22 was denoted as hap^E22^. The results showed that the genotypes of the highlighted SNPs of 14 QTL differed between parents. The results of the haplotype analysis are presented in box plots (**Figure** **6**a). An intriguing discovery was made through haplotype analysis. In hap,^3‐79^ the DRC for fiber quality (DRC_FS, DRC_FUHML, DRC_FU) was greater than that in hap^E22^. Conversely, the DRC for yield (DRC_LP) was greater than that for hap^E22^ compared to that for hap^3‐79^ (Figure 6a).

The higher the DRC_trait is, the stronger the drought tolerance. The haplotype with a higher DRC was selected as the drought‐resistant haplotype for the aggregate analysis of drought QTL in each material within the CSSL. The results showed that there are 5 lines aggregating 14 different drought‐resistant haplotypes in the CSSL (Figure 6b, Table S6, Supporting Information). Moreover, we discovered that the more drought‐resistant haplotypes that the variety aggregated, the greater their MFVD, which indicated the higher drought resistance of the lines (Figure 6c). Concurrently, we examined the yield and fiber quality of 60 lines (30 each) in the two aforementioned extreme mixing tanks. The box plot displayed greater yield traits (LW, BW, and EBN) for the drought‐sensitive lines than for the drought‐resistant lines under drought treatment (Figure 6d). Similarly, the fiber quality (of FUHML, FU, and FS) exhibited comparable trends (Figure 6e). There was no difference in fiber quality between drought‐resistant and drought‐sensitive lines under normal conditions. Nevertheless, following drought treatment, a distinct discrepancy surfaced, with fiber quality deteriorating in drought‐sensitive lines, while drought‐resistant lines displayed greater fiber. Based on the results of the above polymerization analysis and the different advantages of the parent materials in terms of drought resistance, yield, and fiber quality, we believe that the infiltration of genotype 3–79 breaks the phenomenon of linkage and leads to the joint improvement of drought resistance, yield, and fiber quality.

## 3 Discussion

### 3.1 The Necessity and Complexity of Genetic Research on Drought Resistance in Cotton

The drought resistance of plants is regulated by polygenes and is constrained by environmental conditions both internally (physiological status of plants, root structure and function, and leaf characteristics) and externally (weather conditions and soil characteristics). In view of this situation, a series of indicators have been proposed to identify the drought resistance of plants during long‐term treatments, including growth and development morphological characteristics (root characteristics, plant architecture, yield, etc.)^[^ ^34^, ^35^ ^]^; physiological and biochemical properties (plant water potential, water use efficiency, photosynthesis, abscisic acid, proline, etc.)^[^ ^14^, ^36^, ^37^ ^]^; and the comprehensive evaluation of drought resistance (drought resistance coefficient, drought resistance index, membership function value of drought resistance, drought sensitivity index, etc.).^[^ ^38^, ^39^, ^40^ ^]^ However, drought resistance in crops is complex, as crops respond to water differently during different growth periods, and the internal mechanisms of resistance to drought stress are diverse.^[^ ^41^, ^42^ ^]^ Therefore, it is necessary not only to combine morphological, physiological, biochemical, and yield data but also to comprehensively assess drought resistance during each growth period to improve the reliability of identifying drought resistance.^[^ ^36^, ^43^, ^44^ ^]^ In this study, we calculated the DRC to determine the effects of drought stress treatment on the accessions and used the MFVD to determine the DRC of different traits to evaluate the drought resistance of the accessions. Through the MFVD analysis and drought resistance grading of different materials, we screened a series of germplasm resources with strong drought resistance, excellent fiber quality, and high yield that can be directly used as breeding resources for future cotton drought resistance breeding research.

The precision of phenotypic data is decisive in the accuracy of QTL mapping, especially for drought resistance,^[^ ^45^ ^]^ and obtaining accurate and effective phenotypic data controlling the interference of environmental factors has always been a problem. In this study, we obtained meteorological data for Shihezi in Xinjiang during the cotton growth period, and the results showed that the mean monthly temperature and rainfall did not vary much across different years and that the rainfall was lower during the drought treatment period (June‐July) (Figure S4, Supporting Information). Moreover, the mean evaporation in Shihezi is as high as 1500 mm, accounting for 79% of the annual evaporation in summer and autumn.^[^ ^46^ ^]^ The experimental plots undergo annual intensive farming of uniform soil; therefore, external environmental conditions such as rainfall have little impact. Fourteen phenotypic datasets were accurately measured over many years in this population, and the BLUP was used to synthesize phenotypic values to reduce the influence of environmental factors. However, there are also problems associated with large workloads and heavy tasks, and further development of new phenotypic acquisition methods is important for future phenotyping, such as the use of a high‐throughput automated phenotyping platform and high‐throughput field automated phenotyping platform to obtain accurate phenotypic data by means of digital imaging and near‐infrared spectroscopy.^[^ ^47^, ^48^, ^49^, ^50^ ^]^

### 3.2 The Introgression of *G. barbadense* Segments Coordinates the Yield and Quality of *G. hirsutum* Under Drought Conditions

As two of the most important tetraploid cultivated species, *G. hirsutum* and *G. barbadense* exhibit distinctive traits; *G. hirsutum* is known for its high yield and strong adaptability, whereas *G. barbadense* boasts excellent fiber quality.^[^ ^2^, ^3^, ^27^ ^]^ It has been challenging to analyze the disparities between two cotton species and harness their respective strengths to develop cotton varieties with combined high yield and superior fiber quality. Research has shown that long‐term gene flow between *G. hirsutum* and *G. barbadense* enriches the genetic background of these two species, being crucial in the genetic improvement of cotton crops.^[^ ^31^, ^51^ ^]^ Therefore, hybridization and backcrossing were used to artificially promote gene exchange between the two cotton cultivars, after which the excellent main allele of *G. barbadense* was introduced into *G. hirsutum* to overcome the bottleneck of synergistic improvement in cotton resistance, yield, and fiber quality and to create varieties with the advantages of *G. barbadense* and *G. hirsutum*.^[^ ^5^ ^]^ In previous studies, the focus was primarily on using introgression from *G. barbadense* to improve the yield or quality of *G. hirsutum*, and most of the results show a negative correlation between yield and fiber quality. CSSL populations are considered ideal for studying the genetic basis and performing artificial introgression to investigate complex traits by eliminating background noise.^[^ ^5^, ^52^, ^53^ ^]^ Multiple studies have indicated that CSSLs population can break the linkage drag between traits and achieve synergistic improvement.^[^ ^54^, ^55^ ^]^ In this study, we selected 3–79 (with long fiber length and high fiber strength) as the paternal parent and E22 (with high yield and strong drought resistance) as the maternal parent. A population of 319 accessions was constructed using exogenous chromosome fragment infiltration and recombination methods. We employed GWAS, linkage analysis, and BSA strategies, coidentifying 66 QTL for four yield and four fiber quality traits, comprising 39 QTL (identified by at least two strategies) and 6 QTL (identified by all three strategies). Combining the sources of the drought‐resistant haplotypes led to the identification of 14 colocalized QTL. Among these, the genetic infiltration of 3–79 superior loci led to enhanced fiber quality in 13 QTL under drought conditions. One QTL was associated with the improvement of LP under drought conditions beginning at E22. By analyzing the aggregation effects of the 14 QTL with superior loci, we observed substantial enhancements in drought resistance, yield, and fiber quality in the germplasm with the cumulative aggregation of these advantageous loci. Moreover, this method facilitated the screening of a series of accessions that exhibited robust resistance to drought, high yield, and exceptional fiber quality.

### 3.3 Identification and validation of Drought‐Resistant QTL in the CSSL Population Using Multiple Strategies

In this study, a total of 121 significant QTL were identified by GWAS, linkage analysis, and BSA; these QTL were most likely the major QTL affecting drought resistance in cotton. GWAS identified a total of 52 QTL (Table S4, Supporting Information). Linkage analysis identified 34 QTL (Table S4, Supporting Information). Notably, the QTL intervals identified under control conditions differed from those identified using DRC (Figure S5, Table S7, Supporting Information). In addition, the QTL identified in this study were compared to previously reported QTL for drought resistance, with multiple QTL located within or near previously reported drought resistance markers. Li et al.^[^ ^25^ ^]^ used 316 *G. hirsutum* accessions to locate a significant SNP (A05:89717711) on the A05 chromosome, which was also in the QTL (q.DRC_FS.A05.2) interval of GWAS and linkage analysis. The drought resistance QTL (qDSW‐A11‐3) overlapped with the other QTL (q.DRC_FUHML.A11.1, q.DRC_EBN.A11.1, and q.DRC_FS.A11.1) identified in this study at the location of the reference genome.^[^ ^56^ ^]^ Guo et al.^[^ ^57^ ^]^ used 188 *G. hirsutum* varieties for association analysis to identify a significant drought resistance SNP (TM59677) on chromosome D06, which was located within the QTL (q.DRC_BW.D06.1) interval of the GWAS in this study. These results also indicate the reliability of the QTL identified in this study.

In this study, we identified 93 putative candidate genes, many of which are involved in the plant stress response and tolerance. For example, the AT3G02880 (*KIN7*) gene was the most closely related homologous gene to Ghir_D09G017400 in *Arabidopsis*, and *kin7* mutants were more sensitive to drought stress than the wild type in *Arabidopsis*.^[^ ^58^ ^]^ The Ghir_D09G017090 gene encodes a member of the PYR (pyrabactin resistance)/PYL (PYR1‐like)/RCAR (a regulatory component of ABA receptor) family of proteins, which as ABA signaling sensors are widely involved in signaling of stomatal closure, water use and drought stress in *Arabidopsis*.^[^ ^59^ ^]^ Among them, we conducted a functional analysis of two genes, *DRR1*, which encodes a small heat shock protein, and *DRT1*, which encodes a PLETHORA‐like transcription factor, using genetic analysis and CRISPR/Cas9 and VIGS technologies either to verify the function of the genes and haplotypes, which demonstrated the regulatory roles of *DRR1* and *DRT1* in drought stress in cotton and moreover the superior drought resistance of haplotype hap^3‐79^ compared to haplotype hap^E22^ for *DRR1* and *DRT1*. It was suspected that *DRR1* may exert a protective effect against drought stress through its potential chaperone activity, thus reducing damage.^[^ ^60^ ^]^ The homologous gene of *DRT1* in *Arabidopsis* AT3G20840 regulates the growth and cell differentiation of root meristems^[^ ^61^, ^62^ ^]^ and it is regulated by other meristem regulatory factors, such as MERISTEM DEFECTIVE (*MDF*), which maintains meristem activity under environmental stress conditions.^[^ ^63^ ^]^ This suggests that *DRR1* may enhance cotton's drought resistance by maintaining root meristem activity, root growth, and water supply, which need further clarified.

## 4 Conclusions

In summary, an interspecific CSSL population constructed using E22 and 3–79 was used to identify QTL and genes related to cotton yield and fiber quality under drought stress through analysis strategies such as GWASs, linkage analysis, and BSA. Concurrently, CRISPR/Cas9 and VIGS were used to reveal the biological functions of the candidate genes *DRR1* and *DRT1* in drought resistance. Moreover, through the analysis of multilocus aggregation analysis and MFVD, the relationships between drought resistance and yield and between drought resistance and fiber quality were explored, and numerous available drought‐resistance materials were identified.

## 5 Experimental Section

### Plant Materials and Drought Treatments

A population of 319 accessions (Table S8, Supporting Information) derived from a CSSL population generated by crossing and backcrossing “3–79′ (*G. barbadense*) with ‘Emian22” (*G. hirsutum*) was selected for use in a drought treatment experiment on the CSSL population at the flowering and boll stages both in the field in Shihezi, Xinjiang, China (44°18′ N, 85°58′ E) during 2018 and 2019. The construction of this CSSL population was comprehensively outlined in the study conducted by Zhu et al.^[^ ^33^ ^]^ The field experiments were conducted in accordance with a completely randomized block arrangement with two replicates each year. The CSSLs with parents were planted in a block 2.200 m long, 0.900 m wide, and 1.980 m^2^ in area in double rows for each replicate. The plants in the control panel were normally irrigated ten times (5589 m^3^ ha^−1^) during the growth period. The drought treatment was conducted by stopping drip irrigation from the middle of June to late July during the flowering and boll stages (3210 m^3^ ha^−1^) (Table S9, Supporting Information). The planting area was covered with plastic film mechanically on demand, the plant spacing was 10 cm, the actual number of harvested plants was 165 000 plants ha^−1^, and the other aspects of field management were the same as those used in conventional fields. The mean monthly rainfall and temperature in Shihezi were measured during the cotton growing period (Figure S4, Supporting Information).

### Phenotypic Evaluation

The agronomic traits, yield traits and fiber quality of cotton plants were investigated in terms of cotton germplasm resources traits description criteria formulated during the whole growth period.^[^ ^64^ ^]^ FT and WGP were investigated and recorded as important periods for cotton growth. Four indices, namely PH, FFSH, FFSBN, FSBN, and EBN, were investigated through the selection of ten plants of identical growth in late September. Thirty bolls were harvested from the upper, middle, and lower parts for indoor seed testing after boll opening. The three yield trait indices, BW, LW, and LP, were tested for each replicate of each variety. Fiber quality traits, including FUHML, FU, MV, and FS, were determined for 14 g of lint by HFT9000 (temperature: 20 °C, relative humidity: 65%).

The phenotypic data were analyzed using BLUPs via the R package lem4.^[^ ^65^ ^]^ The CV indicated the degree of dispersion between the accessions, *CV* = standard deviation/arithmetic mean. The drought resistance of the accessions was expressed by the DRC (*DRC* = treatment measurement value/control measurement value) of each index.^[^ ^13^, ^40^ ^]^ The drought resistance classification was evaluated by analyzing the membership function value of drought resistance (MFVD) based on yield (BW, LW, LP, and EBN) and fiber quality (FUHML, FS, MV, and FU).^[^ ^36^, ^40^ ^]^ Statistical analyses, significance analyses, and Pearson correlation were conducted using IBM SPSS Statistics 23.0 software (SPSS, Inc., Chicago, IL, USA). Histogram plots, box plots, and violin plots were constructed using R to show the phenotypic distribution.

The Pn and WUE were measured using the functional leaves (every fourth leaf from the top) of the main stems via a Li‐COR 6800 (Li‐6800, LI‐COR, Inc., NE, USA) in the morning (10:30‐12:30 a.m. in Xinjiang) on consecutive sunny days.^[^ ^66^, ^67^ ^]^ The drought resistance of the cotton plants was assessed by relative electrical conductivity and MDA content.^[^ ^68^ ^]^ Leaves from the same part of different single plants were sampled and quickly transferred to a tube filled with 50 mL of distilled water, after which the air in the cell spaces of the leaves was removed by vacuum drying. After 20 min of standing, all the samples were shaken on a shaker at 100 rpm for 3 h at room temperature, and the initial electrical conductivity (C1) was measured by a conductivity meter (DDS‐12A type, Shanghai, China). All the samples were incubated in a boiling water bath for 0.5 h, removed, and cooled to room temperature, after which the conductivity (C2) was measured. The relative electrical conductivity was calculated as *REC* = *C*1/*C*2 × 100.^[^ ^66^, ^69^ ^]^ The MDA was measured following the instructions provided by Suzhou Keming Biotechnology Co., Ltd. The absorbance values at A532 and A600 for both the control and measurement tubes were measured using an Enspire multifunctional microplate reader, and the difference was calculated as Δ*A* = A532 – A600, according to the formula: MDA content (nmol/g fresh weight) = 25.8 × Δ*A*/fresh weight.^[^ ^70^ ^]^

### Genotyping, Mapping, and SNP Calling

The 321 resequenced accessions (319 accessions and 2 parents) were downloaded from the National Center for Biotechnology Information (NCBI), and the software Sentieon was used to call the single nucleotide polymorphism/insertion–deletion (SNP/InDel) with TM‐1 as the reference genome.^[^ ^27^, ^33^, ^71^, ^72^ ^]^ The remaining high‐quality markers were filtered with a MAF ≤  5% and a missing value  ≥ 50% using VCFtools software and were subsequently subjected to genetic analysis.^[^ ^73^ ^]^ Functional annotation of genetic variants was performed by the software ANNOVAR.^[^ ^74^ ^]^

### Genome‐Wide Association Study and Linkage Analysis

For the 14 traits of DRC used for GWAS, a mixed‐model approach was adopted using the factored spectrally transformed linear mixed models (FaST‐LMM) v2.07, with 1309780 SNPs across the entire cotton genome.^[^ ^75^ ^]^ The suggestive and significant association threshold values were set at *p*‐value (1/SNP) = 7.63487E^−07^ and a *p*‐value (0.05/SNP) = 3.81743E^−08^. Manhattan plots were created using the R software package qqman. A linkage disequilibrium (LD) block heatmap containing the SNP loci (−log_10_(7.63487E‐^07^) ≥ 6.11) associated with phenotypes was generated using LDBlockShow to show significant haplotype structures and determine the candidate interval.^[^ ^76^ ^]^ The introduced fragments of each accession were redivided using the bin map constructed in the early stage of the laboratory, and the genotype file of linkage analysis was obtained (Table S10, Supporting Information). QTL mapping was performed using the RSETP‐LRT‐ADD mapping method and the default parameters of QTL IciMapping V4.2 software.^[^ ^77^ ^]^

### BSA‐seq Analysis

MFVD was used to screen 60 extreme phenotypic samples from the CSSL population, comprising 30 drought‐resistant and 30 drought‐sensitive lines, and two extreme mixed pools (drought‐resistant pool and drought‐sensitive pool) were constructed. For these pooled samples, high‐quality DNA was extracted individually from each sample, and after equal mixing, the pools were subjected to BSA‐seq. With the use of Sentieon software, the SNP variants were called by employing TM‐1 as the reference genome for the pooled dataset.^[^ ^27^, ^72^ ^]^ The pooled data were filtered using the genotypes of the recurrent parent (E22) and nonrecurrent parent (3‐79). The QTL intervals were identified by employing the R package QTLseqr to calculate the ΔSNP‐index between two pooled samples within a 1.5 Mb window.^[^ ^78^ ^]^ The QTL were named starting with “q”, followed by an abbreviation of the phenotype trait, the chromosome number, and the number of QTL affecting traits on chromosomes. The results of GWAS, linkage analysis, BSA‐seq, and RNA‐seq analysis of cotton drought stress were identified, and the genes with differential expressions within the QTL interval of the candidates and the functional annotation associated with the regulatory pathways of drought stress as candidate genes were identified. Previously published RNA‐seq data were obtained for a drought‐resistant line (M307) and a drought‐sensitive line (M048) of CSSLs.^[^ ^66^ ^]^

### RNA Extraction and RT‐qPCR Analysis

Total RNA was extracted using a HiPure HP Plant RNA Mini Kit (Magen Biotech Co., Ltd.) according to the manufacturer's instructions. RNA integrity was confirmed by agarose gel electrophoresis, and RNA concentration and mass were measured with a NanoDrop 2000 spectrophotometer (Thermo Fisher Scientific, US). RT‒qPCR was performed using an ABI 7500 system (Applied Biosystems, Foster City, CA). The relative expression level of each gene in three biological replicates was calculated by the 2^−(ΔΔCt)^ method, and *GhUBQ7* (GenBank: No.DQ116441) was used as an internal reference for other genes in cotton.^[^ ^79^ ^]^

### Transgenic Vector Construction and Genetic Transformation

The vectors for *DRR1* (Ghir_A11G032300) and *DRT1* (Ghir_D09G012870) were constructed using established CRISPR/Cas9 gene editing processes.^[^ ^80^ ^]^ The genetically constructed vector, derived from the *Agrobacterium tumefaciens* electrocompetent strain GV3101, was utilized for genetic transformation. The cotton cultivar Jin668, known for its high genetic transformation efficiency, was used as the recipient material. The specific steps for cotton genetic transformation were described previously.^[^ ^81^ ^]^ Mutants were detected using established Hi‐TOM processes.^[^ ^82^ ^]^ The primers used are listed in supplementary TableS11–1 (Supporting Information).

### VIGS Assay

The candidate genes were subjected to a VIGS assay to verify the function of the genes in M048 and M307. The VIGS assay involves two major vectors, *pTRV:1*, and *pTRV:2*. The fragments of the genes 5′ untranslated region (300‐500 bp) were cloned and ligated into the *pTRV2* vector, which was subsequently electroporated into the *Agrobacterium tumefaciens* strain GV3101. The *pTRV:2* vector without the target gene was used as a control and was named TRV:*00*. *GhCLA* (TRV:*CLA* [CLOROPLASTOS ALTERADOS1]), a key enzyme in chloroplast synthesis, was selected as an efficiency indicator. The VIGS procedure was described previously.^[^ ^83^, ^84^ ^]^ The plants were subsequently grown in a growth room at 26–28 °C with a 16 h photoperiod. The young leaves of each gene were harvested for RNA extraction and silencing level determination when plants inoculated with TRV:*CLA* exhibited a photobleaching phenotype. The plants in the drought treatment group were not watered at the two‐leaf stage, while those in the control group were watered every four days. The plants were photographed, and physiological indices were detected until the soil volumetric water content decreased to ≈10% under drought treatment. The soil volume moisture content was determined by AM150 (Delta‐T Devices Ltd., UK). The primers used are listed in supplementary Table S11–2,3 (Supporting Information).

### Statistical Analysis

The sample size and statistical methods for each figure in the study are denoted in the legends. Student's *t*‐test or Duncan's multiple comparisons test were used to evaluate the statistical significance between samples in bar graphs and box plots. All error bars represent SD, and significance was denoted as (*) *p*‐value < 0.05, (**) *p*‐value < 0.01, and (***) *p*‐value < 0.001, ns indicates no significant difference.

## Conflict of Interest

The authors declare no conflict of interest.

## Author Contributions

X.Y. and X.Z. conceived and designed the experiments. B.H. conducted the experiments and wrote the manuscript. The CSSLs were generated by Z.L. Y.M., Y.Y., and Y.W. assisted with field cultivation, management, and phenotypic collection. P.Y. and Z.L. provide assistance in the creation of transgenic materials. W.Z., F.W., D.Y., and B.Z. assisted in the collection of phenotypic data. All authors had read and approved the final manuscript.

## Supporting information

Supporting Information

Supplemental Table 1

Supplemental Table 2

Supplemental Table 3

Supplemental Table 4

Supplemental Table 5

Supplemental Table 6

Supplemental Table 7

Supplemental Table 8

Supplemental Table 9

Supplemental Table 10

Supplemental Table 11

## Data Availability

The data that support the findings of this study are available in the supplementary material of this article.
