# Supplementary material for: Dissecting the Superior Drivers for the Simultaneous Improvement of Fiber Quality and Yield Under Drought Stress Via Genome‐Wide Artificial Introgressions of Gossypium barbadense into Gossypium hirsutum
Source: Adv Sci (Weinh). 2024 Jul 10;11(34):2400445. doi: 10.1002/advs.202400445 (PMC11425955; doi:10.1002/advs.202400445)
Supplement: Supplementary file 1 — Supporting Information [file ADVS-11-2400445-s009.docx]

**Supporting Information:**

Dissecting the Superior Drivers for the Simultaneous Improvement of Fiber Quality and Yield under Drought Stress Via Genome-Wide Artificial Introgressions of *Gossypium barbadense* into *Gossypium hirsutum*

*Bei Han, Wenhao Zhang, Fengjiao Wang, Pengkai Yue, Zhilin Liu, Dandan Yue, Bing Zhang, Yizan Ma, Zhongxu Lin, Yu Yu, Yanqin Wang, Xianlong Zhang, Xiyan Yang**


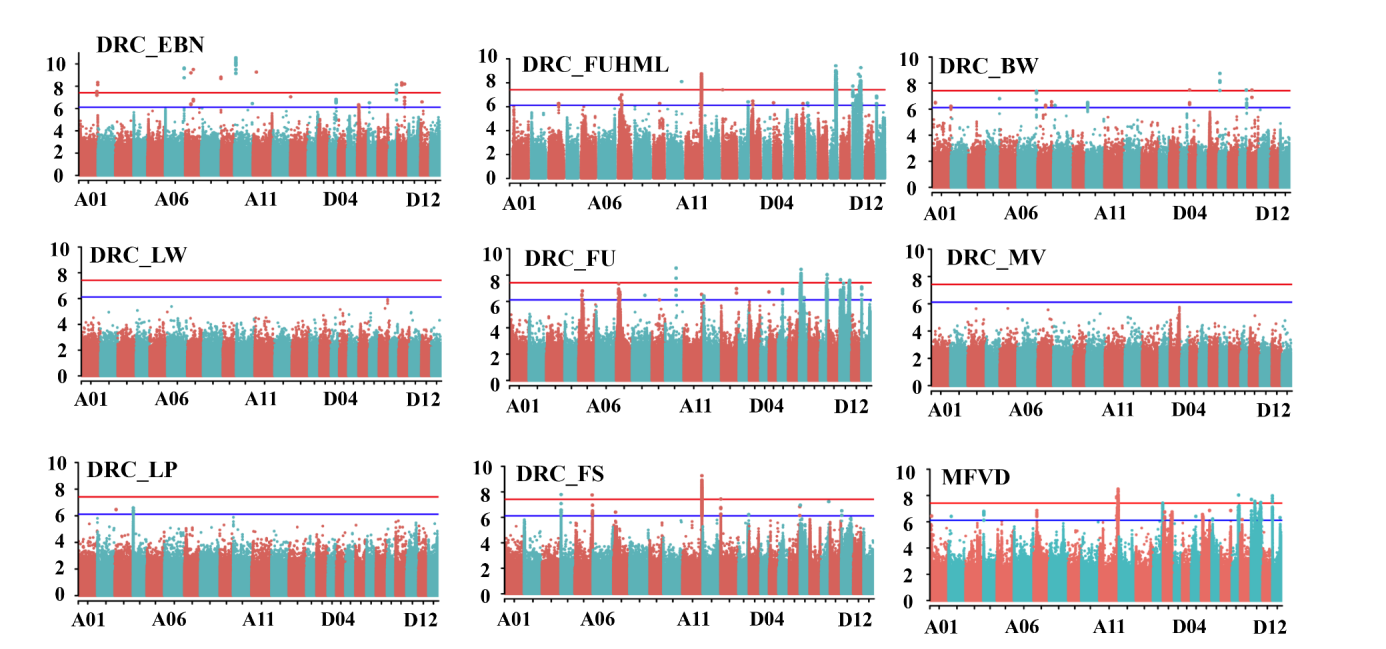


**Figure S1 The GWAS results for 8 DRC phenotypes and MFVD in CSSLs populations.**

**
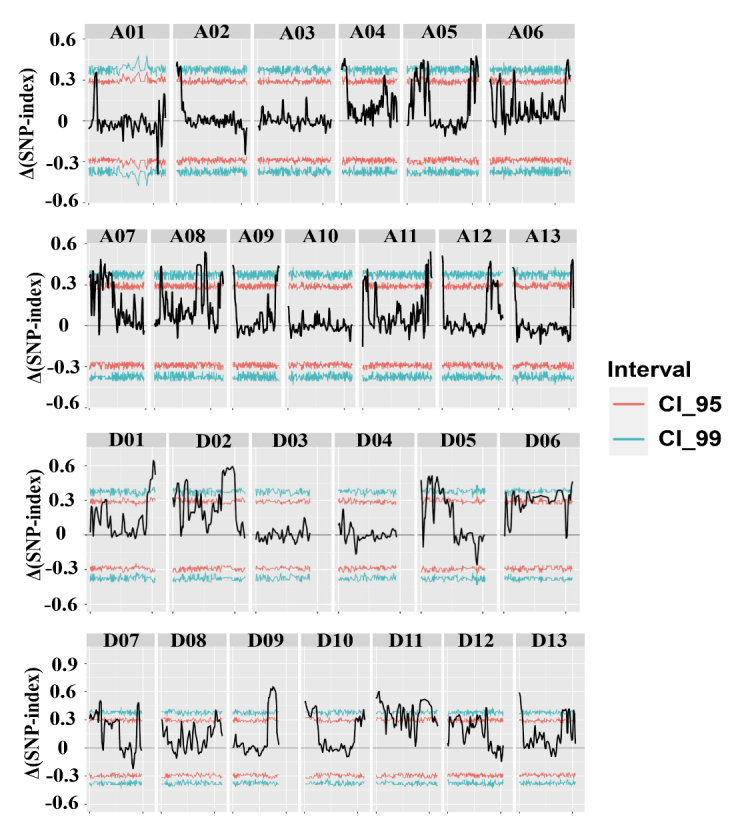
**

**Figure S2 Data from the bulk segregation analysis (BSA) were used to identify genomic regions affecting cotton drought tolerance.** Each solid black line represents the fitted ΔSNP-index value, while the red and blue lines correspond to 0.95 and 0.99 thresholds of the ΔSNP-index, respectively.

**
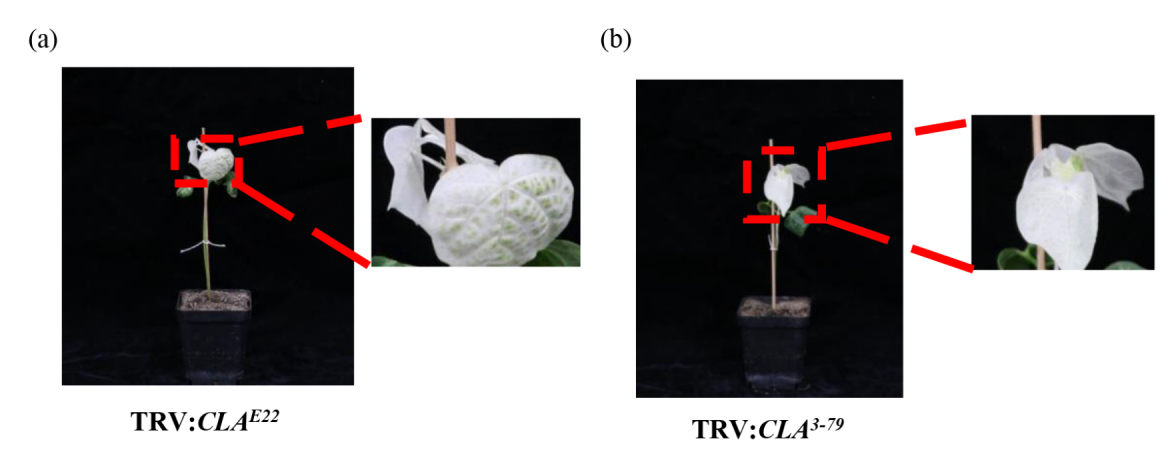
**

**Figure S3 The true leaves of TRV:*CLA*-expressing plants showed a photobleaching phenotype in M307(a) and M048 (b).**


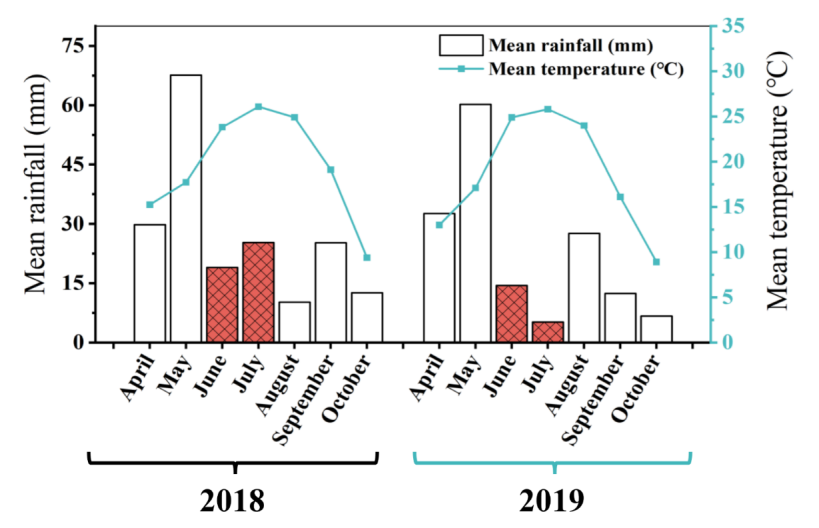


**Figure S4 Meteorological data of the whole growth period of cotton in Shihezi, Xinjiang, 2018 – 2019.**

**
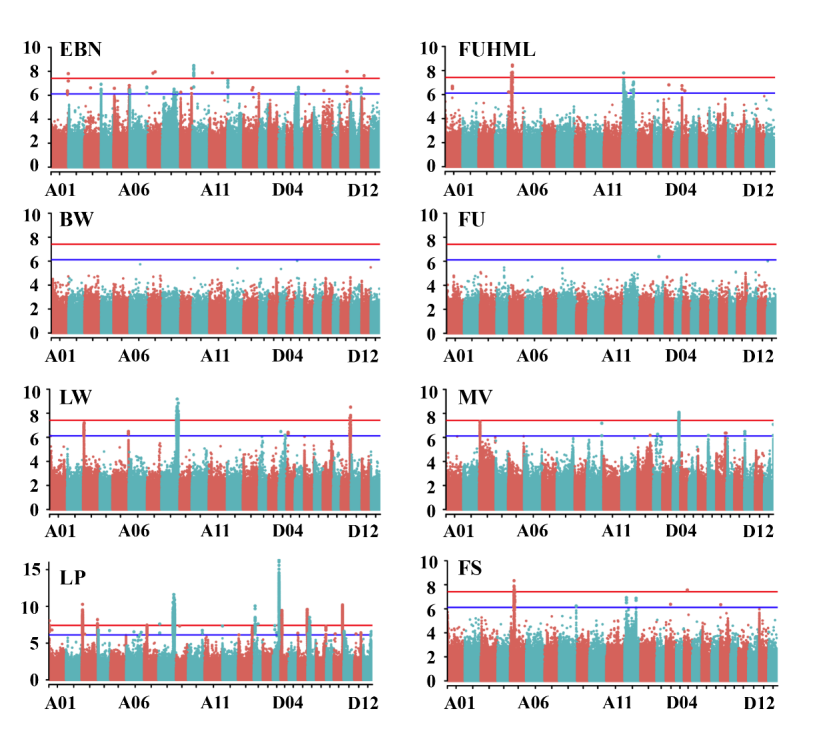
**

**Figure S5 The GWAS results for 14 phenotypes in CSSLs populations under control conditions.**
